# Correction: Validation of postnatal growth and retinopathy of prematurity (G-ROP) screening guidelines in a tertiary care hospital of Pakistan: A report from low-middle income country

**DOI:** 10.1371/journal.pone.0314483

**Published:** 2024-11-21

**Authors:** The PLOS ONE Editors

## Notice of Republication

This article [1] was republished on May 17, 2024, to address an issue identified post-publication.

In addition, the article [1] was republished on November 12, 2024, to correct errors in the author list. The updated author list as follows:

Haroon Tayyab^co^, Adnan Mirza^co^, Roha Ahmad Choudhary*, Hassan Jabbar*, Mohammed Abbas Motiwala*, Sehrish Nizar Ali Momin*, Shiraz Hashmi*, Khadijah Abid^co^

^co^ Contributed equally to this work: Haroon Tayyab, Khadijah Abid, Adnan Mirza.

* RAC, HJ, MAM, SNAM, SH also contributed equally to this work.

The updated citation is: Tayyab H, Mirza A, Choudhary RA, Jabbar H, Motiwala MA, Momin SNA, et al. (2024) Validation of postnatal growth and retinopathy of prematurity (G-ROP) screening guidelines in a tertiary care hospital of Pakistan: A report from low-middle income country. PLoS ONE 19(5): e0302534. https://doi.org/10.1371/journal.pone.0302534

The article’s Data Availability statement is updated to: Underlying data is available from the corresponding author on request and may be provided with a future update to this article. Any other information related to data are available from the assistant manager in the department of ophthalmology and visual sciences, AKUH, Karachi, Pakistan. Email: asif.hukma@aku.edu.

Please download this article again to view the correct version.
